# Storstrømmen and L. Bistrup Bræ, North Greenland, Protected From Warm Atlantic Ocean Waters

**DOI:** 10.1029/2021GL097320

**Published:** 2022-03-14

**Authors:** Eric Rignot, Anders Bjork, Nolwenn Chauche, Ingo Klaucke

**Affiliations:** ^1^ Department Earth System Science University of California Irvine Irvine CA USA; ^2^ Jet Propulsion Laboratory California Institute of Technology Pasadena CA USA; ^3^ Department Geoscience and Natural Resources University of Copenhagen Copenhagen Denmark; ^4^ Access Arctic Liernais France; ^5^ GEOMAR Helmholtz ‐ Centre for Ocean Research Kiel Germany

**Keywords:** Greenland, bathymetry, sea level, ocean, Storstrømmen

## Abstract

Storstrømmen and L. Bistrup Bræ are 20‐ and 10‐km wide, surge type glaciers in North Greenland in quiescent phase that terminate in the southernmost floating ice tongue in East Greenland. Novel multi‐beam echo sounding data collected in August 2020 indicate a seabed at 350–400 m depth along a relatively uniform ice shelf front, 100 m deeper than expected, but surrounded by shallower terrain (<100 m) over a 30‐km wide region that blocks the access of warm, salty, subsurface Atlantic Intermediate Water (AIW) at +1.6°C. Conductivity temperature depth data reveal waters in front of the glaciers at −1.8°C not connected to AIW in the outer fjord, Dove Bugt. The recent grounding line retreat of the glaciers is attributed to glacier thinning at its ablation rate, with little influence of ocean waters, which illustrates the fundamental importance of knowing the bathymetry of glacial fjords.

## Key Points

- Storstrømmen Glacier, Greenland (27‐cm sea level rise equivalent) stands in cold waters behind shallow bathymetry
- The frontal regions are 100 m deeper than expected but access of warm Atlantic Intermediate Water (AIW) is limited by a wide and shallow ridge
- Retreat of Storstrømmen and L. Bistrup Bræ glaciers has operated relatively independent of changes in AIW temperature in Dove Bugt

## 1 Introduction

The Greenland Ice Sheet has been losing an increasing amount of mass in the last few decades, from 41 ± 17 Gt/yr in 1990–2000 to 286 ± 20 Gt/yr in 2010–2018 (Mouginot et al., 2019). During that time period, Storstrømmen Glacier, a major outlet part of the Northeast Ice Stream in Greenland, has been undergoing surges for an overall minimal contribution to the total mass loss (Mouginot et al., 2018). Its average surface mass balance decreased from +2 Gt/yr in the 1970s–1980s, to +0.7 Gt/yr in the 1990s, and −0.3 Gt/yr in the 2000s–2020s (Mouginot et al., 2019). Its 58,176‐square‐km basin, the fifth largest in Greenland, holds enough ice to raise sea level by 27 cm, in contrast to 56 cm for Zachariae Isstrøm and 60 cm for Nioghalvfjerdsfjorden, the other two components of the Northeast Ice Stream (An et al., 2021; Schaffer et al., 2020). Storstrømmen is grounded below sea level at the ice front but rises above sea level at the junction with Zachariae. The glacier meets with the L. Bistrup Bræ Glacier at the ocean boundary to form the southernmost floating section in east Greenland (Rignot et al., 2001). The drainage of Storstrømmen reaches the summit of Greenland (Reeh et al., 2003). The glacier surges every 50–70 years, with the last surge occurring in 1978–1984 (Reeh et al., 1994).

A leading hypothesis for the evolution of Greenland glaciers is that the enhanced intrusion of warm Atlantic Intermediate Water (AIW) along the coast melted the ice fronts, de‐stabilized the glaciers, and increased mass discharge (Catania et al., 2019; Christoffersen et al., 2011; Holland et al., 2008; Howat et al., 2008; Murray et al., 2010; Wood et al., 2021). In the absence of bathymetry and oceanographic data, however, it has been noted that it is challenging to interpret the observed glacier evolution (Wood et al., 2021). The International Bathymetric Chart of the Arctic Ocean version 3.0 (IBCAO Ver. 3.0) and BedMachine Greenland V3 (BMv3) do not include quality bathymetry data in Dove Bugt and in the frontal regions of Storstrømmen or L. Bistrup Bræ because they have never been surveyed (Jakobsson et al., 2012), making it impossible to assert if subsurface (>350 m depth) AIW reaches the glaciers.

Starting in 2015, NASA’s Ocean Melting Greenland (OMG) Earth Venture Suborbital mission (Fenty et al., 2016) collected multi‐beam echo sounding (MBES) data by ship, airborne high resolution gravity data, airborne surface topography, and air‐launched Airborne eXpendable Conductivity, Temperature and Depth (AXCTD) probes and traditional ship‐based Conductivity, Temperature, Depths (CTDs) (An et al., 2019; Wood et al., 2018). The data have been used to reconstruct glacier thickness and bed elevation from mass conservation in BMv3 (Morlighem et al., 2017) and combined with new bathymetry data in IBCAO Ver. 4.0 (Jakobsson et al., 2020). The data product provides a smooth and reliable transition in bed elevation at the glacier fronts, which is critical for ice sheet numerical models. The mapping effort provided a major revision of glacier depths, with bed elevations many 100 m deeper than previously thought in many fjords. Greater water depth in the glacial fjords means greater exposure to AIW, more efficient undercutting of the glacier calving margins by the ocean, and stronger response to climate warming.

Here, we present the results of the first oceanographic survey of Storstrømmen and L. Bistrup Bræ conducted in the summer of 2020 with MBES and CTD data along their ice fronts, and CTD data combined with remote sensing going back to the 1960s and 1970s. We employ the results to interpret the recent glacier evolution. We conclude on the role of the ocean in melting this major glacier setting.

## 2 Data and Methods

**Figure 1:**
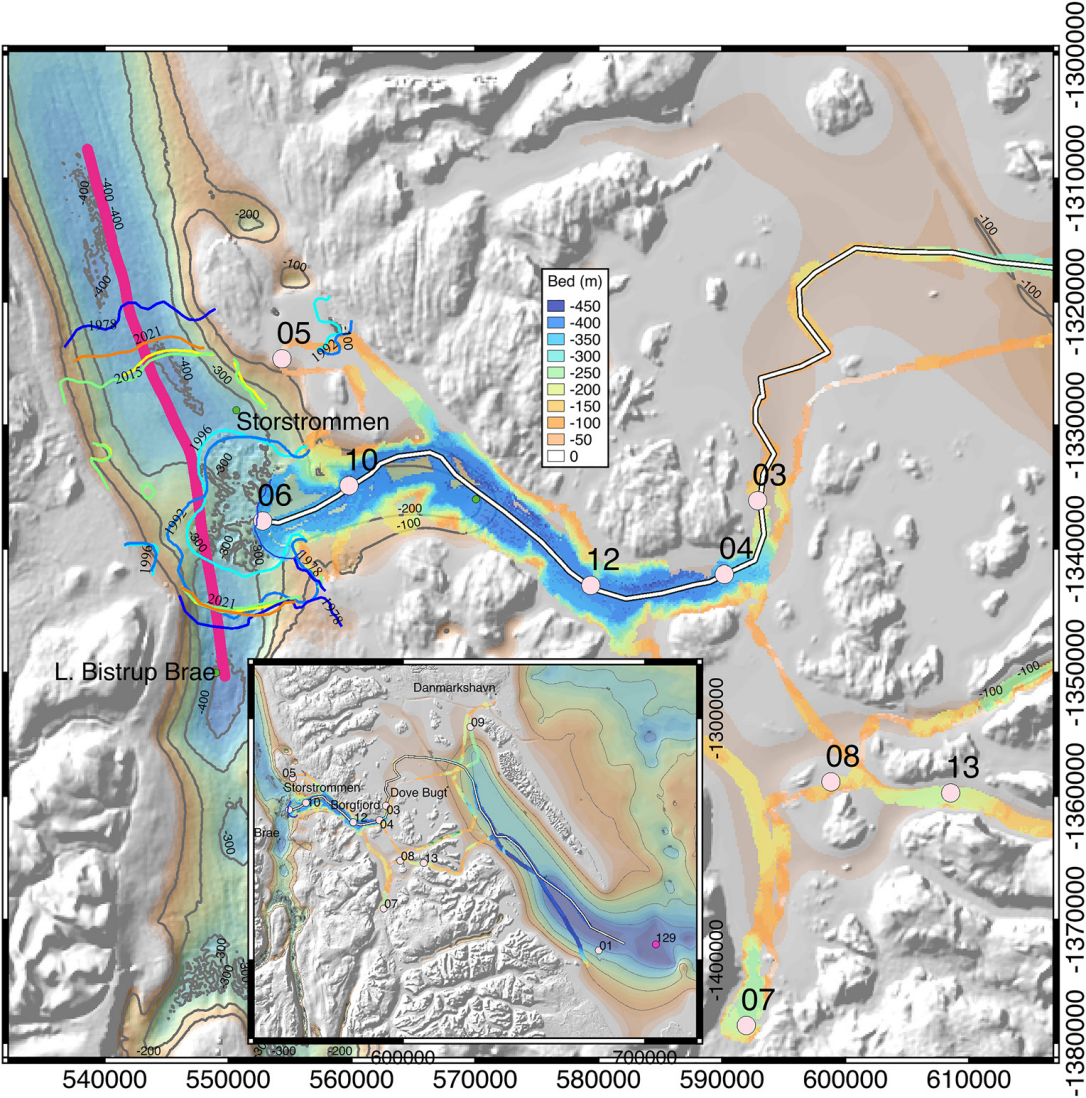
Storstrømmen and L. Bistrup Bræ glaciers, Greenland, ending in Borgfjorden with grounding line positions from 1978 (dark blue), 1992 (light blue), 1996 (cyan), 2015 (light green), 2017 (yellow) (Mouginot et al., 2018), and 2021 (orange), 100 m‐bed contours from BMv3 in black, Conductivity, Temperature, Depth (CTD) casts from August 2020 as light pink circles with black labels, and bed elevation from multi‐beam echo sounding data color coded from 0 to 450 m depth. Pathline used in Figure 2 is white. Radar sounding line used in Figure 5 is thick purple on the left hand side of the figure. Inset shows broader area and the location of all CTD data in Figure 2. Coordinates are eastern UTM 27°N EPSG 32627.

*Background.* The 21‐km wide Storstrømmen (i.e., “Large Stream”) Glacier (Weidick et al., 1994) drains into Borgfjorden and connects with Dove Bugt, near the Danish outpost of Danmarkshavn, east of Dronning Louise Land. Discovered in 1906 by Ludvig Mylius‐Erichsen, the glacier is confluent with the equally large (16 km) L. Bistrup Bræ Glacier from the south (Reeh et al., 1994; Weidick et al., 1994) (Figure 1). Storstrømmen—flanked by stagnant Kofoed‐Hansen Bræ to the north—drains a sector 58,176 km^2^ in area, with a 273 mm sea level equivalent. L. Bistrup Bræ—flanked by small AD Drachmann to the east—drains 22,306 km^2^ with a volume of ice above flotation equivalent to 62 mm of sea level rise. Storstrømmen retreated 15 km in 1913–1950, remained stable in 1950–1978, and re‐advanced by 8 km in 1978–1984 during a surge (Reeh et al., 1994). The grounding line of Storstrømmen advanced by 14 km in 1978–1992, remained stable in 1992–1996, and retreated by 10 km (400 m/yr) in 1996–2017. The grounding line of L. Bistrup Bræ advanced by 1.5 km in 1978–1992, 3.5 km in 1992–1996, and retreated by 3.5 km in 1996–2017. At present, the ice shelf area of Storstrømmen is 201 versus 53 km^2^ for L. Bistrup Bræ.

The recurrence cycle of the surges observed between 1978 and 1984 has been estimated at 50–70 years (Mouginot et al., 2018; Reeh et al., 1994). The early Holocene recession has brought Storstrømmen to its present position (Weidick et al., 1994). L. Bistrup Bræ surged in 1988, with a recurrence estimated at 30–50 years. The upstream part of Storstrømmen thickened 20 m in 1994–2014, while the lower elevation region thinned 30 m. Accounting for the rapid ice discharge in 1978–1984, Storstrømmen has been estimated to be close to a state of mass balance over one surge cycle (Mouginot et al., 2018). For L. Bistrup Bræ, the same calculation suggested a glacier below a state of mass balance but there is also less complete information its surge cycle. At present, the ice discharge into the ocean of the glaciers is at the 0.2 and 0.1 Gt/yr levels, respectively.

*Grounding Line Retreat.* The grounding line was first mapped in 1992 and 1996 using ERS‐1 data (Rignot et al., 2001) and updated with Sentinel‐1a/b in 2015–2017 (Mouginot et al., 2018). Here, we update the grounding line position to November 2021 using a triplet of Sentinel‐1a/b radar interferometry data acquired on 16, 28 October, and 9 November along Track 170, Frame T083607 (Figure 1).

**Figure 2:**
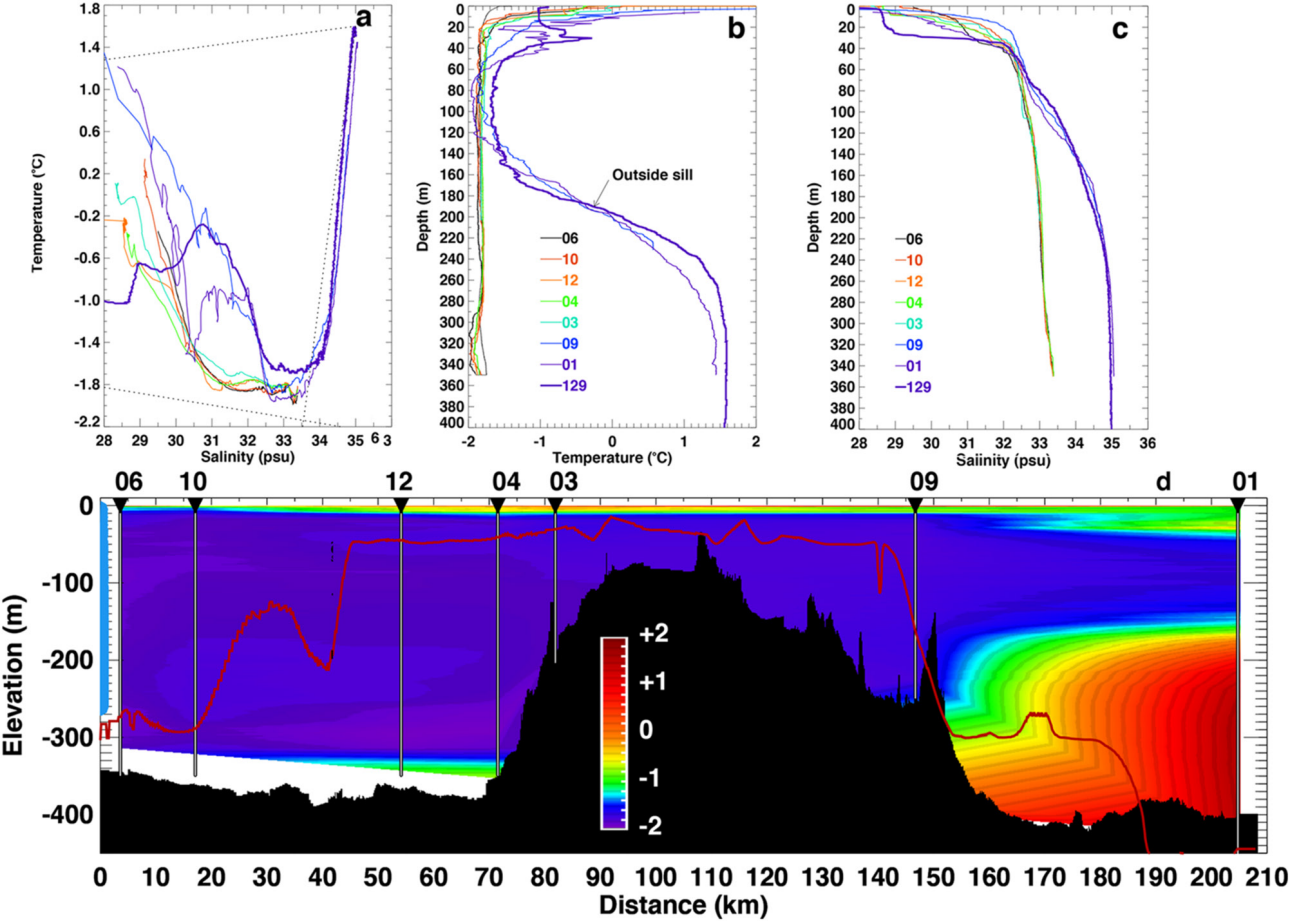
Storstrømmen Glacier, Greenland (a) temperature and salinity for Conductivity, Temperature, Depth (CTD) casts in Borgfjorden (03, 04, 06, 10, 12) (see Figure 1) and outside the sill (09, 01, 129); (b) temperature and (c) salinity versus depth color coded by casts. Black dotted lines in (a) are the freezing point of seawater at 300 m depth (bottom), mixing of Atlantic Intermediate Water (AIW) and runoff (top), and mixing of AIW with ice melt (Gade’s curve on right Gade, 1979). (d) Bathymetry and ocean temperature along the white pathline in Figure 1 color coded from −2°C (blue) to +2°C (red) with seafloor in black, and bed elevation from BMv3 in thin red line. CTD casts are marked as thin white vertical lines. The floating ice face of Storstrømmen Glacier is colored light blue on left with a cavity entrance between 260 and 350 m depth. Regions with no temperature data are white.

**Figure 3:**
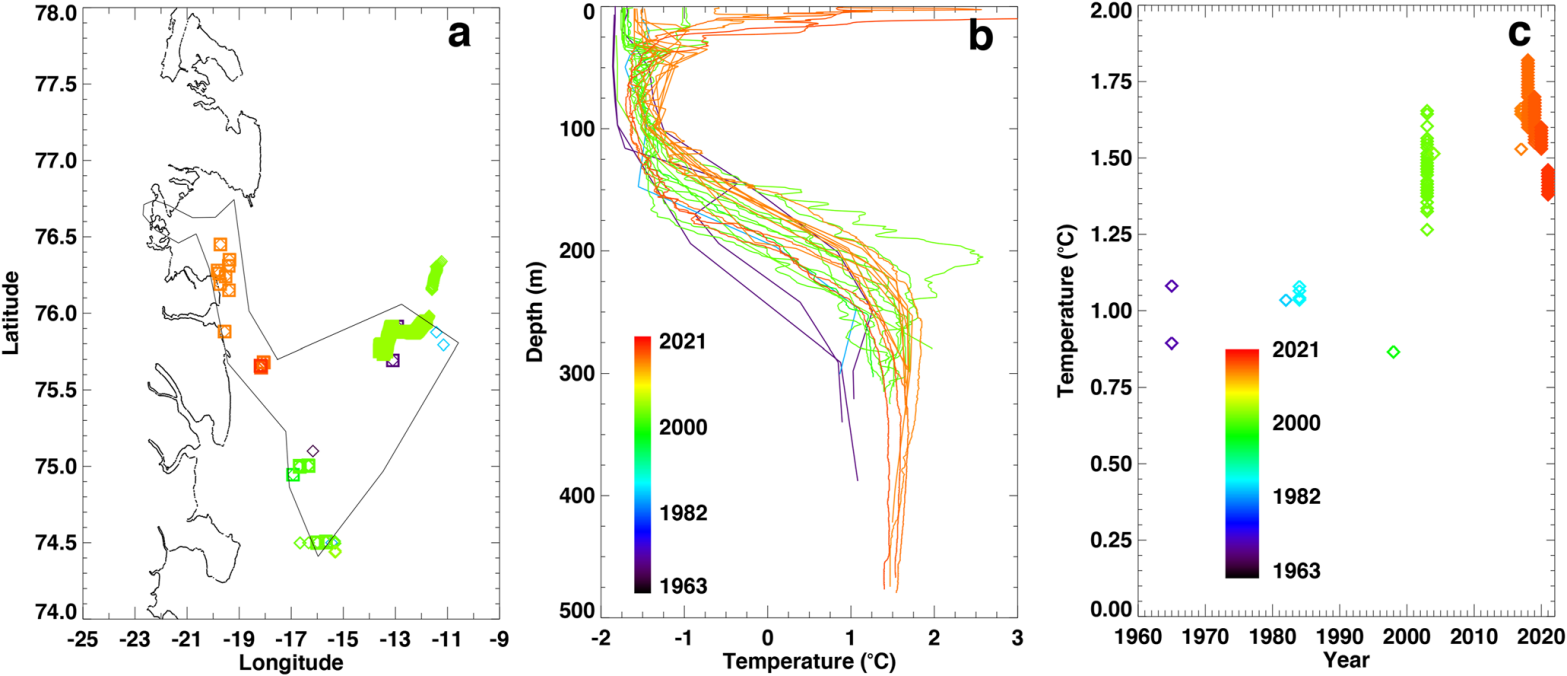
Conductivity, Temperature, Depth (CTD) data in the broader Dove Bugt, Greenland with (a) CTD location color coded from 1963 to 2021, coastline is black, and CTD domain is thin black; (b) potential temperature color coded from 1963 to 2021 versus depth; and (c) change in ocean temperature between 300 and 400 m depth.

*Ocean Thermal Forcing.* Air‐launched AXCTD data were collected on a Grumman Gulfstream III aircraft in September/October 2016, C‐130 Hercules in October 2017, a Basler DC‐3 Turbo Prop in August/September 2018, and a Basler BT‐67 in August/September 2019, 2020 and 2021. During the ship survey, we collected traditional ship‐launched CTD with an Applied Microsystems Ltd (AML) Minos‐X probe. We combine the AXCTD data with historical CTD data from the Hadley center (bodc.ac.uk) spanning from 1960 to 2017 to document temperature changes in Dove Bugt. We analyze the CTD data in front of the glaciers (Figure 2) and in Dove Bugt (Figure 3).

**Figure 4:**
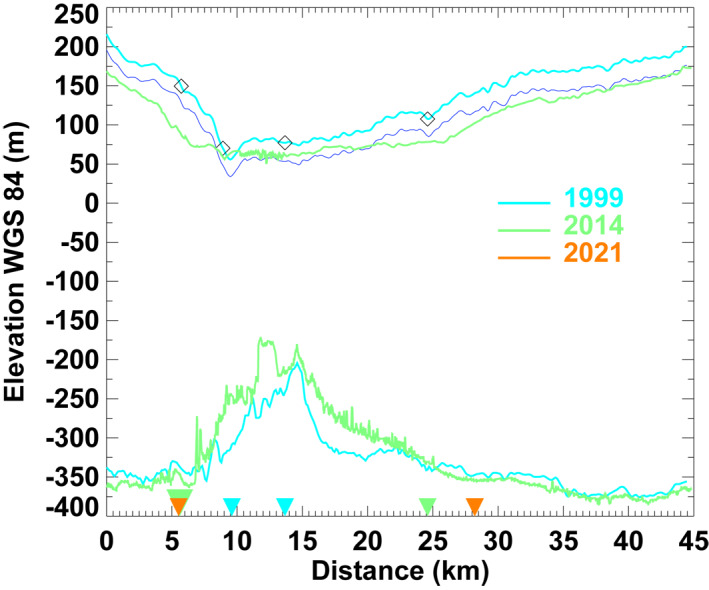
Surface (top) and bed (bottom) elevation recorded on 19 May 1999 (cyan, as Figure 1) and on 29 April 2014 (green) by MCoRDS (Gogineni, 2012) from L. Bistrup Bræ (left) to Storstrømmen Glacier (right) along profile colored in purple in Figure 1. Grounding lines are marked as triangles in 1996 (cyan), 2015 (green) and 2021 (orange). Ice thinning, surface and bed slopes are calculated in between the open diamonds symbols. The 1999 surface elevation updated with surface mass balance is colored blue.

*Ice Thinning.* We employ two lines of radar sounding data collected on 19 May 1999 and 29 April 2014 by the Multichannel Coherent Radar Depth Sounder (MCoRDS) (Gogineni, 2012) running approximately along the glacier center lines (Figure 1). We calculate ice thinning, surface slope and basal slope on grounded ice over 4‐km segments in 1999–2014 (Figure 4). Surface (bed) slope is positive if the surface (bed) elevation drops in the flow direction.

*Bathymetry.* We collected MBES data onboard the M/V Wave in August 2020 using the deep water (>1,000 m) GEOMAR Helmholtz Centre for Ocean Research Kiel's Elac SeaBeam 1050, 50 kHz multibeam echo sounder (MBES) which we combined with an interferometric swath sonar for the shallower water (<350 m), the ITER‐SYSTEM’s Bathyswath‐2 117 kHz. The Elac sonar was mounted on the stern of the boat 0.8 m below the water level whereas the Bathyswath‐2 was mounted on the hull of the M/V Wave at an angle of 42° from the vertical. Correction for sound velocity (range calculation and ray‐tracing) is performed using real‐time sound velocity sensor mounted on the head of the transducers and CTD data collected at regular intervals (An et al., 2018) from an AML Oceanographic Minos X CTD. The Qinsy QPS and Cloud Compare softwares are used to process the MBES data from both sonars. The bathymetry data is processed onto a regular grid at 25 m spacing (Figure 1).

In BMv3, mass conservation was applied to reconstruct glacier thickness (Morlighem et al., 2017) using Operation IceBridge ice thickness data, a velocity map from 2007 to 2008 (Rignot & Mouginot, 2012), surface mass balance data for 1961–1990 (Noël et al., 2019), ice thinning data (Csatho et al., 2014), and constrained by multibeam echo sounding data where available (Fenty et al., 2016). We do not expect the ice front depth to match the measured bathymetry given the paucity of radar thickness data in the area and glacier stagnation.

## 3 Results

The grounding line of on Storstrømmen retreated 1.1 km from 2017 to 2021, or at a rate of 250 m/yr since 2015 (Figure 4). The grounding line of L. Bistrup Bræ did not retreat in 2015–2021. For Storstrømmen, we calculate an ice thinning of 23 ± 5 m from the radar‐derived thickness data from 1999 to 2014 immediately above the region of grounding line retreat, a bed slope of −0.91%, and a surface slope of 0.28%, which yields a grounding line retreat from hydrostatic equilibrium of 13 ± 3 km versus 11 ± 0.5 km observed. For L. Bistrup Bræ, surface slope is 2.4%, bed slope is −0.71%, ice thinning is 45 ± 5 m (Figure 4), which yields 2 ± 0.2 km calculated retreat versus 3 ± 0.5 km observed. Within uncertainties, we conclude that the grounding line retreat is reasonably well explained by ice thinning alone.

In addition, we estimate the cumulative thinning from surface mass balance in 1999–2014 (Noël et al., 2019). We deduce 23 ± m of thinning on grounded ce, which agrees with the observed thinning (Figure 4), which confirms that the glacier thins at its ablation rate. Over recently ungrounded ice, we find no additional thinning, that is, ice shelf rates are low.

**Figure 5:**
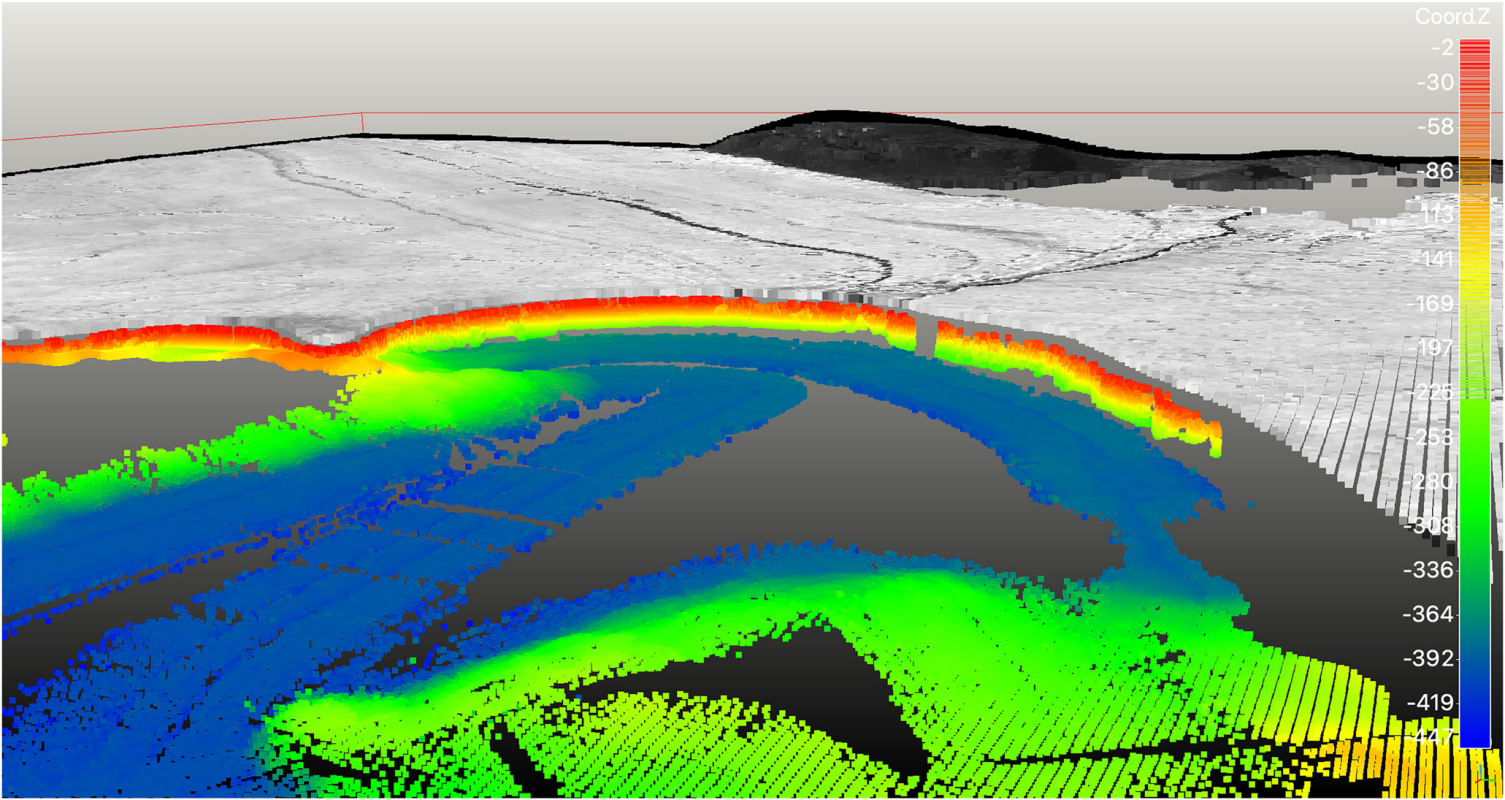
Perspective view (looking to the northeast) with 1:1 scale of the bathymetry in front of Storstrømmen Glacier color coded from −450 m (blue) to 0 m (red) with ice face, cavity under ice face (i.e., ice shelf), overlaid by a Landsat‐8 image from August 2020 with surface elevation from Greenland Ice Sheet Mapping project. Areas with no data are shaded gray.

The ITER Bathyswath‐2 imaged the submerged glacier face of Storstrømmen and L. Bistrup Bræ (Figure 5). Echoes are recorded within a few meters below the water line until a depth of approximately 230–250 m. Beyond that depth, we record no sonar echo until a seafloor depth of about 380 m. This data record reveals the entrance of an ice shelf cavity, with a water column height of about 130–150 m (Figure 5). The presence of this cavity is consistent with the presence of an ice shelf. Indeed, the sonar echoes penetrate the cavity and image the sea floor depth several hundred meters beyond the ice shelf front. At the ice shelf front, the revised bed elevation is 120 ± 20 m lower than in BMv3, which is not surprising given the paucity of prior bed data in the lower reaches of the glacier. The result of this survey will therefore help refine BMv3.

In Dove Bugt, warm, saline water of Atlantic origin (1.6°C, 35 psu) is found below 250 m depth (Figure 2). No CTD data prior to 2021 exist in front of Storstrømmen and L. Bistrup Bræ. We detect a warming between 300 and 400 m depth in the 2000s versus the 1980s (Figure 3). The warming persists until 2019, before a slight cooling in 2020 and 2021. Overall, ocean water temperature increased by +0.7 ± 0.2°C since the 1960s–1980s at that depth. In the shallower waters (<100 m depth), we detect no warming.

In front of the glaciers, water temperature is cold at −1.8°C almost uniformly from 20 to 380 m depth. The data contrasts with the temperature in Dove Bugt at +1.6°C. The ocean waters are also fresher in front of the glaciers, with a salinity at 33.5 psu. In the temperature/salinity plot, the CTD data follow Gades' ice melt line between 400 and 200 m depth, then the curve converges toward the freezing point of seawater (Figure 2), before migrating toward the runoff line at shallower depths.

As the glacier does not terminate in a calving cliff, but as a floating ice shelf, it is difficult to assess the impact of the ocean waters on ice melt. Assuming that the undercutting model developed for a vertical face is also applicable at the glacier grounding line of Storstrømmen, we calculate the rate of undercutting as in Rignot et al. (2016). The rate of undercutting, *q*_*m*_, depends on subglacial water discharge, water depth, and thermal forcing, $q_{m}=\left(0.0003\;b\;q_{sg}^{0.33}+0.15\right)TF^{1.18}$, where *b* is water depth in meters, *q*_*m*_ and *q*_*sg*_ are in meters per day, and TF is thermal forcing. With a freezing point of −2.1°C for seawater at 380 m depth at a salinity of 33.5 psu (Figure 4), thermal forcing, TF, is 0.3°C, which yields *q*_*m*_ = 0.04 m/d or 13 m/yr in the absence of runoff. This rate of undercutting is one order of magnitude lower than the observed retreat of the glacier grounding line (250–380 m/yr). The ocean is too cold to yield much glacier undercutting.

The ocean warming in Dove Bugt of 0.7°C ± 0.2°C is less than the 1.3°C ± 0.5°C warming for Zachariae Isstrøm and Nioghalvfjerdsfjorden between 240 and 500 m depth, but comparable to the 0.85°C ± 0.2°C warming at 300–400 m depth for Humboldt. Due to the presence of broad sills.

## 4 Discussion

A wide region of shallow ground, with numerous islands, protects Storstrømmen and L. Bistrup Bræ from the influence of warm AIW waters at present. The bathymetry reveals an over‐deepening in Borgfjorden, where the two glaciers merged in earlier times and most likely yielded enhanced basal erosion, but the overdeepening does not extend more than 46 km past the current ice front, hence does not form a natural, deep passage for AIW to reach the glaciers from Dove Bugt. Although a number of deep passages are detected in between the islands, we find no natural passage for AIW to enter the inner fjord, as confirmed by the CTD data.

The grounding line retreat is well explained by ice thinning at its ablation rate. The same conclusion applies to Soranerbren Glacier, a glacier immediately southeast of L. Bistrup Bræ (bottom part of Figure 1), which is also protected from warm waters by shallow ground. This is important because these two glaciers are among the few large glaciers in Greenland that are naturally protected from the intrusion of AIW.

In a quiescent phase, Storstrømmen accumulates mass—and possibly subglacial water at depth—in the upper reaches of the terminal valley, where the glacier is thickening (Reeh et al., 1994). Once a threshold is reached and water pressure exceeds the ice overburden pressure, strong basal motion will displace large amounts of ice downstream. The absence of strong melt by the ocean waters at the glacier junction may contribute to the surge type nature of Storstrømmen. Larger rates of basal ablation by the ocean at the ice front would reduce basal resistance, enhance glacier flow, lubricate the bed, and reduce the likelihood of building surge cycles. Other surge‐type glaciers in North Greenland are in a similar configuration. The ice shelf front of Hagen Bræ is buttressed by a few islands (Joughin et al., 2010; Rignot et al., 2001; Solgaard et al., 2020). AXCTD data from 2020 reveal a fjord depth of 170 m (AXCTD 100B) with a bottom ocean temperature of −0.5°C, that is, AIW does not penetrate the fjord. Academy Glacier, farther north, another surge type glacier (Joughin et al., 2010), has a frontal depth of 275 m, with a bottom water temperature of +0.3°C (AXCTD 2021 100C), hence Academy Glacier is also relatively protected from warm waters.

Among the largest 226 glaciers that control 96% of the ice discharge of the Greenland Ice Sheet, it has been noted that 87 glaciers have not been surveyed for bathymetry and ocean temperature and as a result remain undiagnosed in terms of their recent evolution (Wood et al., 2021). Storstrømmen, L. Bistrup Bræ, Soranerbren, Kofoed‐Hansen Bræ, and A.D. Drachmann, were five of these previously unknown glaciers, with a significant reserve of ice above sea level in their drainage basins. In retrospect, it would have been difficult to interpret the steady mass balance of these glaciers with ongoing ocean warming in Dove Bugt if we did not have a detailed knowledge of the fjord bathymetry and ocean temperature in front of the glaciers. It therefore remains essential to survey the remained glaciers.

## 5 Conclusions

In this study, we present new bathymetry data for two major outlet glaciers of East Greenland, Storstrømmen and L. Bistrup Bræ. The revised sea floor is 130 m deeper than previously thought, but we find no bathymetric passage for warm, salty AIW to travel from the (deeper) outer fjord (Dove Bugt) to the (shallower) glacier fronts. At the glacier fronts, the ocean temperature is only −1.8°C, which does not yield much bottom melt of the ice shelf. For Storstrømmen and L. Bistrup Bræ glacier, we are also able to interpret their grounding line retreat as almost entirely caused by ice thinning. In contrast to most other large outlet glaciers in Greenland, the ocean waters do not play a strong role in the evolution of Storstrømmen and L. Bistrup Bræ glaciers in a warming climate.

## Data Availability

CTD and multibeam echo sounding data are readily available from https://omg.jpl.nasa.gov/portal/data/OMGEV-AXCTD/ and https://omg.jpl.nasa.gov/portal/data/products/urn:omg:OMG_Bathy_MBES_L1. BMv3 is available at https://nsidc.org/data/IDBMG4/versions/3.
